# Robust Keypoint Detection and Matching on Fisheye Images by Self-Supervised Learning

**DOI:** 10.1155/2022/4024774

**Published:** 2022-12-22

**Authors:** Wei Tian, Pei Cai, Yongkun Wen, Xinning Chu

**Affiliations:** School of Automotive Studies, Tongji University, 201804 Shanghai, China

## Abstract

Accurate image feature point detection and matching are essential to computer vision tasks such as panoramic image stitching and 3D reconstruction. However, ordinary feature point approaches cannot be directly applied to fisheye images due to their large distortion, which makes the ordinary camera model unable to adapt. To address such a problem, this paper proposes a self-supervised learning method for feature point detection and matching on fisheye images. This method utilizes a Siamese network to automatically learn the correspondence of feature points across transformed image pairs to avoid high annotation costs. Due to the scarcity of the fisheye image dataset, a two-stage viewpoint transform pipeline is also adopted for image augmentation to increase the data variety. Furthermore, this method adopts both deformable convolution and contrastive learning loss to improve the feature extraction and description of distorted image regions. Compared with traditional feature point detectors and matchers, this method has been demonstrated with superior performance on fisheye images.

## 1. Introduction

In recent years, visual feature extraction and keypoint matching have been widely applied in computer vision tasks, such as motion and behavior analysis [1, 2] and visual localization [3], which are essential to autonomous driving vehicles. In autonomous driving perception tasks, the traditional way to obtain environmental information is to use a narrow-angle pinhole camera, which yet has a limited field of view (FOV), and thus leads to a large range of blind spots. On the one hand, when the camera pose changes, the limited viewing angle can lead to the loss of feature points. On the other hand, the small FOV of the narrow-angle pinhole camera can be easily occupied by dynamic vehicles and pedestrians, resulting in incorrect pose estimation.

**Figure 1:**
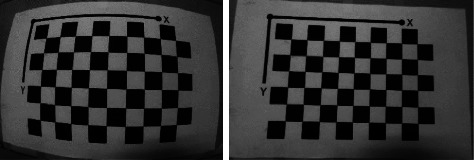
The significant visual distortion of the fisheye image (a) and compared to that of the standard image (b).

In contrast, the fisheye camera can perceive a wide range of a scene, and even obtain visual information about the hemispheric domain theoretically [4].  Figure 1 shows the visual difference between fisheye images and standard images. The middle part of the fisheye image protrudes and the part on the image boundary is compressed, leading to significantly varied resolution across the image. This distortion characteristic is a particular challenge for vision tasks such as keypoint matching and object detection. Standard images are with a consistent resolution and look closer to the real world. Usually, fisheye images should be rectified before applying conventional image-processing algorithms.

**Figure 2:**
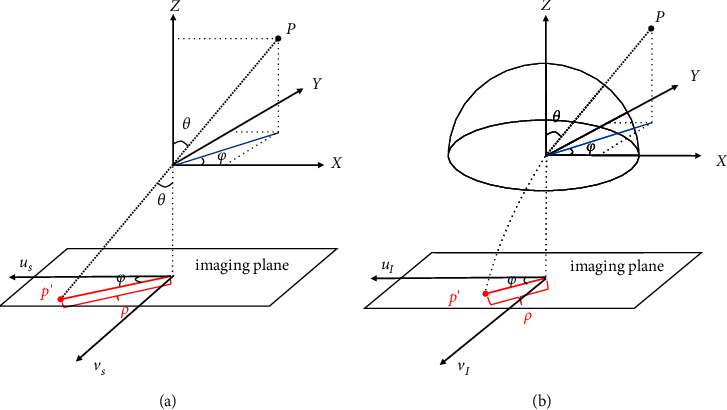
Projection models of the pinhole camera (a) and fisheye lens camera (b).

The large distortion in the fisheye image is attributed to the unconventional fisheye lens, which corresponds to a nonlinear projection as shown in Figure 2. In the pinhole projection model, the perspective projection of a point **P** from the 3D camera coordinate system *X*-*Y*-*Z* to the imaging plane *u*_*s*_-*v*_*s*_ (denoted as *u*_*I*_-*v*_*I*_ in the fisheye model) can be simply formulated by

(1) $$\begin{array}{c} \rho =f\cdot \tan\;\theta \text{,} \end{array}$$

where *ρ* denotes the distance between the projected point **p**′ on the imaging plane and the optical axis while *f* is the focal length. The angle of incident light is denoted as *θ*. However, the nonlinear projection of a fisheye lens is more complex and can be expressed by different mathematical models [4] according to the design and manufacturing, such as stereographic projection, equidistance projection, equisolid angle projection, and orthogonal projection, respectively, interpreted as follows:

(2) $$\begin{array}{c} \rho =2f\cdot \tan\frac{\theta }{2}\text{,}\\ \rho =f\cdot \theta \text{,}\\ \rho =2f\cdot \sin\frac{\theta }{2}\text{,}\\ \rho =f\cdot \sin\;\theta \text{.} \end{array}$$

**Figure 3:**
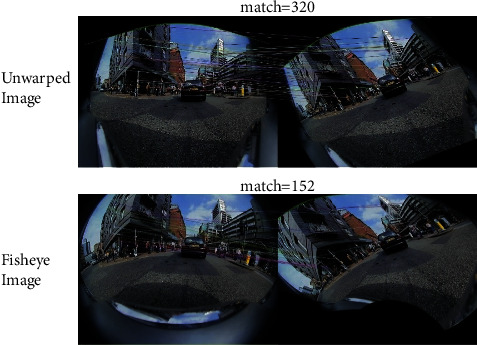
An example of image rotation. The number of matched points by SIFT on unwarped images is 320 (a), while on fisheye images (b), it is only 152, with a reduction of more than a half.

The spatially varying distortion induced by the fisheye lens leads to strong appearance variations of the objects, especially for those in close-by surroundings [5]. Therefore, the processing algorithms for fisheye images are much more sophisticated, which are comparatively underexplored than those on standard images. However, the research about processing fisheye images is of great practical significance, as fisheye cameras have been widely applied in many fields such as navigation, road and tunnel inspection, and video surveillance, with details stated as follows. (1) Navigation: mobile robot navigation with panorama vision is one of the focuses of current researches. The perception module consisting of fisheye cameras can obtain a surround-view perception of the environment at a reduced number of perception sensors, and benefit the subsequent tasks such as trajectory tracking and navigation [6]. (2) Road and tunnel inspection. Health assessments of infrastructures are essential for construction tasks. For surface damage detection with a coverage of 360°, techniques with panorama vision such as fisheye cameras are prevalent [7–9], which helps to avoid serious incidents and thus ensure public safety. (3) Video surveillance: the hemispherical lens is commonly applied in modern surveillance devices [5] to provide a large FOV containing as much information as possible from the monitored environments. Fisheye cameras are also highly favored in tasks related to autonomous driving and 3D reconstruction, where accurate keypoint matching lays a solid foundation for follow-on vision tasks. However, due to significant distortion, general camera models (such as the pinhole model) and ordinary keypoint descriptors cannot be well applied in processing fisheye camera images (Figure 3).

Currently, research works on fisheye images mostly focus on undistortion schemes [10, 11]. In the image registration task, these schemes are utilized to undistort fisheye images, on which the keypoints are extracted and matched. However, the undistortion process in such methods will inevitably give rise to field-of-view loss and resampling artifacts [5]. Let alone, very few pioneer researches have explored keypoint detection and matching, which can directly apply to fisheye images. Additionally, uncertainties or noises in images can also influence the detection. Effective solutions are image preprocessing methods such as fuzzy logic-based ones [12, 13].

To date, keypoint models can be mainly categorized into traditional and deep learning-based methods. Compared to traditional ones, descriptors generated by deep learning can interpret much richer image information. Under the background that deep learning-based methods gradually occupy the mainstream, the research of fisheye images in this field currently encounters the following problems:

- Computer vision algorithms based on supervised learning require large-scale accurately annotated images. However, the scarcity of well-labeled fisheye image datasets limit the development of corresponding image-processing algorithms based on supervised learning.
- The nonlinear projection of the fisheye lens leads to the large distortion of images. Therefore, image-processing algorithms based on the pinhole camera model cannot be directly applied to fisheye images. It is necessary to create algorithms to extract features according to the characteristics of fisheye images.

Considering the problems, we propose a self-supervised learning method for fisheye image keypoint detection and matching, whose performance surpasses the traditional models.

Our contributions are summarized as follows:

- We introduce a keypoint detection and matching approach for fisheye images based on self-supervision within one round of learning
- We present an image transform pipeline to simulate the viewpoint change of fisheye images, which can help the self-supervised learning of keypoint correspondences across images
- We integrate both the deformable convolution and the contrastive learning loss into the network to strengthen the feature learning on fisheye images
- We conduct comprehensive evaluations on the WoodScape fisheye dataset and demonstrate that our method outperforms the baseline, as well as the traditional methods such as SIFT, SURF, ORB, BRISK, KAZE, and AKAZE.

The remainder of this work is organized as follows: Section 2 gives an overview of related work.  Section 3 introduces the fisheye image viewpoint transform scheme, and the self-supervised learning approach for fisheye image keypoint detection and description.  Section 4 shows the experimental results.  Section 5 concludes this work.

## 2. Related Work

Here, research studies related to this work are reviewed in three aspects: (a) handcrafted keypoint models, (b) learning-based keypoint models, and (c) fisheye image undistortion approaches.

### 2.1. Handcrafted Keypoint Models

Traditional feature point detection methods include FAST [14], SIFT [15], SURF [16], ORB [17], KAZE [18], and AKAZE [19]. The FAST is a simple and efficient detector by comparison only with the surrounding pixels [14]. However, it cannot characterize feature points. Unlikely, the SIFT includes a descriptor of local image features that are invariant to rotation, scaling, and brightness changes, and also maintain a stability to a certain extent for angle changes, affine transforms, and noise [15]. However, its computational load is high. The SURF is a simplified version of SIFT with gradient approximation by Haar-like filters [16]. However, its advantages on runtime are still limited. The ORB algorithm is based on the directional FAST feature detection and the BRIEF feature description [17]. KAZE [18] and AKAZE [19] deploy approximations to speed up calculation in nonlinear scales. It enjoys a fast processing speed and can be applied in scenarios with high real-time requirements.

### 2.2. Learning-Based Keypoint Models

Simo-Serra et al. proposed a simple scheme of a Siamese network consisting of two same branches to learn the discriminating representation of a local patch [20]. By mining both positive and negative samples, they achieved high performance in the patch description. The LIFT [21] uses a spatial transformer layer to rectify the image patch for feature point detection, description, and orientation estimation. However, it is trained in multiple steps and requires the supervision from structure from motion (SFM) systems. The QuadNetworks [22] trains CNNs to rank points in a transform-invariant fashion. They can perform both single-modal and cross-modal interest point detection, yet without providing descriptors. The TILDE [23] selects keypoint candidates across multiple images from the same viewpoint to learn regressors, which are robust against drastic image changes by weather and lighting conditions. However, their approach is not explicitly trained for rotation and scaling invariance. The SuperPoint [24] built a self-supervised framework to train both detectors and descriptors for interest points, which are extracted from semidense grids. This method is first trained on synthetic data and then on real images, resulting in two tedious rounds of training. The UnSuperPoint [25] was proposed as an improvement of the SuperPoint. It predicts keypoint locations by regression, and introduces a new loss function to train point detectors within a Siamese architecture in a self-supervised manner. It requires only one round of training and does not require the generation of pseudo ground truth points. Nevertheless, the above methods are mainly applied to pinhole camera images.

### 2.3. Fisheye Image Undistortion

The fisheye image undistortion is to correct distortions of the image induced by the nonlinear characteristics of the lens. The correction process starts from the optical imaging model, and reconstructs the incident ray using the camera parameters obtained by the calibration. Then, it builds a spatial mapping from the spherical perspective projection to the plane (or cylinder) projection [4]. Kannala and Brandt [26] proposed a flexible radially symmetric projection model with circular control points to improve the calibration accuracy. It is easy to expand and versatile and can be applied to cameras of both narrow and wide-angle lenses. Hartley and Kang [27] proposed a new scheme that does not establish any specific distortion model, but calibrates the radial distortion in a parameterless manner. However, this scheme is relative sensitive to noise. Wang et al. [28] proposed an extremely wide-angle camera model which complies with the equidistant projection principles. Based on that, it also gives four calibration methods that can be applied to a variety of application scenarios with high accuracy.

In this paper, we also propose a deep learning-based approach for feature point detection and description. Our approach is based on the UnsuperPoint [25] yet differs from it in three points. Firstly, based on the fisheye image undistortion, we adopt an image transform pipeline for data augmentation which is consistent with the viewpoint change of fisheye images, and thus beneficial for the learning of keypoint correspondences in real scenes. Furthermore, we integrate both deformable convolution and contrastive learning loss to enhance the feature learning on fisheye images, yielding more discriminative keypoint descriptors.

## 3. Proposed Approach

### 3.1. Fisheye Image Viewpoint Transform

**Figure 4:**
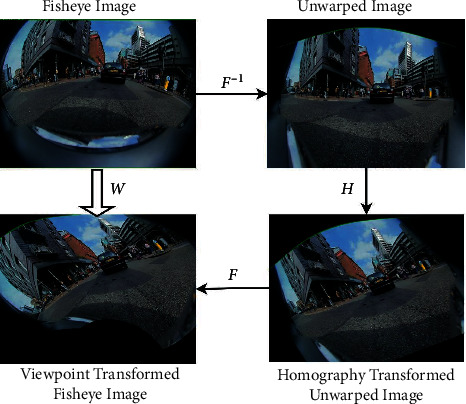
Overview of the fisheye image viewpoint transform.

As in [25], the self-supervised learning of keypoints requires transformed image pairs. However, the direct homography transform used by pinhole camera images cannot be applied to fisheye images due to their nonlinear projection characteristics. Therefore, we adopt a fisheye image viewpoint transform, as shown in Figure 4. The source fisheye image is firstly undistorted according to the projection model. A homography transform is then applied on the unwarped image. After that, the image is further warped into the target fisheye image, which can be considered as the source fisheye image undergoing viewpoint change.

More specific steps about this process are described here: we define the 2D spatial mapping from the fisheye image domain *𝕀*^2^ to the unwarped image domain *𝕊*^2^ as: *ℱ* : *𝕀*^2^⟶*𝕊*^2^. Thus, the inverse operation *ℱ*^−1^ denotes the mapping from the unwarped image domain to the fisheye image domain: *ℱ*^−1^ : *𝕊*^2^⟶*𝕀*^2^. The homography transform of an ordinary image *S* ∈ *𝕊* is denoted as: *S*_*H*_=*ℋ*(*S*). With the operations described, we can generate a new fisheye image *I*′ from the source *I* in following steps:

(3) $$\begin{array}{c} I^{\prime }=W\left(I\right)=F^{-1}\left(H\left(F\left(I\right)\right)\right)\text{.} \end{array}$$

The mapping *ℱ* varies with the undistortion scheme. Through the mapping *𝒲*, we can obtain the paired fisheye images before and after the viewpoint transform. It should be noted that although the method is based on an undistortion scheme, the final output is still a fisheye image.

### 3.2. Image Warping Scheme

Here, we assume both extrinsic and intrinsic parameters of the fisheye camera are given. According to the spherical projection model, pixels on the fisheye images are firstly projected onto the spherical surface of a unit radius. Thus, points can be represented with 3D coordinates in the camera coordinate system. In a further step, the points are converted into the world coordinate system through the camera's extrinsic parameters. After that, the pinhole camera model is used to project the 3D points back to the ordinary image plane coordinates. In this way, the unwarped image after distortion correction can be obtained. Practically, to avoid image sparsity, each pixel on the new image is inversely transformed to the corresponding subpixel position on the original image, and the bilinear interpolation is used for sampling.

In this work, the camera is oriented in the horizontal direction. The image coordinate system is modified by locating its origin at the image center and changing the unit to the meter. Given a pixel with coordinates **p**_*s*_=(*u*_*s*_, *v*_*s*_) on the unwarped image *S*_*H*_, which has undergone the homography transform *ℋ*, we first use the pinhole camera model to project it onto the cylindrical surface and further convert it to a point **P** on a spherical surface with a unit radius. According to [29], its 3D coordinates can be formulated as follows:

(4) $$\begin{array}{c} \left[\begin{array}{c} X\\ Y\\ Z \end{array}\right]=\frac{1}{\sqrt{v_{s}^{2}+f^{2}}}\left[\begin{array}{c} f\;\sin\theta_{s}\\ v_{s}\\ f\;\cos\theta_{s} \end{array}\right]\text{,} \end{array}$$

with *θ*_*s*_=arctan*u*_*s*_/*f*, and *f* denotes the focal length.

**Figure 5:**
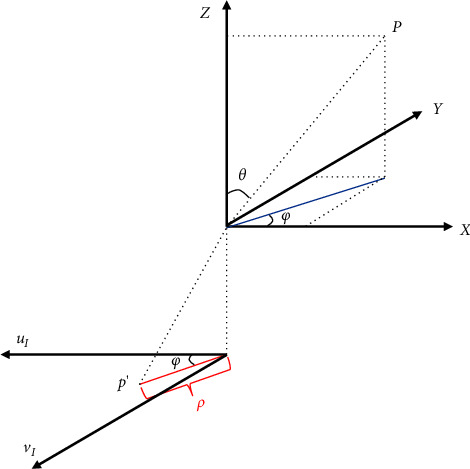
Fisheye camera projection model.

Then, we use the fisheye camera model to project the point from the 3D space back to the image coordinates **p**′=(*u*_*I*_, *v*_*I*_) on the new fisheye image *I*′ [26]. The projection process in the fisheye camera model is shown in Figure 5. The coordinates of point **p**′ can be calculated as follows:

(5) $$\begin{array}{c} \left[\begin{array}{c} u_{I}\\ v_{I} \end{array}\right]=\frac{\rho \left(\theta \right)}{C}\left[\begin{array}{c} X\\ Y \end{array}\right]\text{,} \end{array}$$

with

(6) $$\begin{array}{c} \rho \left(\theta \right)=a_{1}\theta +a_{2}\theta^{2}+\cdots +a_{n}\theta^{n}\text{,}\\ \theta =\arccos\left(\frac{Z}{\sqrt{X^{2}+Y^{2}+Z^{2}}}\right)\text{,}\\ C=\sqrt{X^{2}+Y^{2}}\text{.} \end{array}$$

The coefficients *a*_1_,…, *a*_*n*_ can be provided by the fisheye camera projection model.

### 3.3. Self-Supervised Keypoint Learning

**Figure 6:**
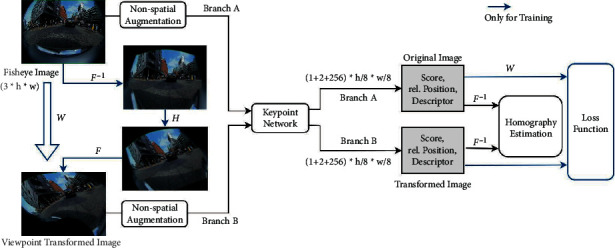
Overview of proposed self-learning architecture. The source fisheye image is firstly transformed into a viewpoint-changed version by undistortion, homography transformation, and warping, respectively. The keypoint network is applied on both source and transformed fisheye images to detect keypoints, interpreted by scores, relative positions, and descriptors. Based on the matching of keypoints, the homography transform between two fisheye images is further estimated and the losses are calculated (during training).

The fisheye viewpoint transform is incorporated into the self-supervised keypoint learning architecture as shown in Figure 6. This architecture utilizes a Siamese structure with a twin of branches. The input of branch *A* is the source image, while for branch *B* it is the viewpoint-transformed version of the source image by mapping *𝒲*. Both images undergo a random nonspatial transform such as color conversion or noising. Thereafter, a shared keypoint network is applied to predict keypoint scores, relative positions, and descriptors on both images. Prediction errors of the two branches are calculated in the loss function to guide the network training.

#### 3.3.1. Keypoint Detection and Description Network

The keypoint detection and description network used in the self-supervised learning architecture is based on the work [16] and its parameters are listed in Table 1. This network consists of a backbone and three output heads. The RGB image is firstly fed into the backbone to generate a small feature map with a size of only 1/8 of the input image. The feature map is further processed by the subsequent heads to output three tensors with the same size, each in the representation of scores, relative positions, and descriptors of keypoints, respectively. As can be seen, each score, relative position, and descriptor in the output corresponds to an 8 × 8 region of the input image.

Since the visual features are nonuniformly scaled due to the distortion on the fisheye image, it will be inappropriate to apply the same convolutions on different image regions. Therefore, we apply the deformable convolution in the keypoint network based on the fact that it has a stronger adaptability than ordinary convolution to complex geometric deformation. Specifically, in the convolutional layers of both backbone and output heads, we adopt the deformable convolution so that the model can better learn the features in the distorted image.

Additionally, for each convolutional layer, the stride is set to 1 and the kernel size equals 3. All convolutional layers are followed by batch normalization and an activation function of Leaky ReLU, except the last layer in each head.

#### 3.3.2. Learning Loss

The learning loss considers the similarity of corresponding points on their positions, scores, and descriptors. Simultaneously, it encourages the spatially uniform distribution, repeatability of feature points, and decorrelation between nonidentical point descriptors, similar to [25]. The total loss can be decomposed into four parts: the self-supervised loss *L*_ssp_, the uniform position distribution loss *L*_uni_, the descriptor correspondence loss *L*_desc_, and the descriptor decorrelation loss *L*_decor_, interpreted as follows:

(7) $$\begin{array}{c} L=\alpha_{ssp}L_{ssp}+\alpha_{uni}L_{uni}+\alpha_{desc}L_{desc}+\alpha_{decor}L_{decor}\text{,} \end{array}$$

where *α*_ssp/uni/ de sc/ de cor_ indicates the corresponding weight.

The self-supervised loss *L*_ssp_ can be further interpreted as follows:

(8) $$\begin{array}{c} L_{ssp}=\alpha_{pos}L_{pos}+\alpha_{score}L_{score}+\alpha_{rep}L_{rep}\text{,} \end{array}$$

where the position loss *L*_pos_ is designed to minimize the Euclidean distance of paired points, thus ensuring that each pair corresponds to the same point in the original image. The score loss *L*_score_ is to ensure an identical score prediction for point pairs, specifically by minimizing the squared score difference. The repeatability loss *L*_*rep*_ is to ensure that paired points with a close distance have a higher score, while pairs of far away points have a lower score. Given the predicted scores *s*_*A*_ and *s*_*B*_ by the twin branches *A* and *B* of the Siamese learning architecture for the *i*-th point pair, the loss *L*_rep_ can be calculated as follows:

(9) $$\begin{array}{c} L_{rep}=\underset{i}{\sum }\frac{s_{A}+s_{B}}{2}\left(d_{i}-\bar{d}\right)\text{,} \end{array}$$

where *d*_*i*_ indicates the distance between the *i*-th paired points, while $\bar{d}$ represents the mean distance of all point pairs.

The loss *L*_uni_ is to ensure a uniform distribution of predicted keypoints within the grid, rather than concentrating on the grid boundary. Thus, it is represented by summed differences between the distribution of predicted point coordinates and a uniform distribution. The loss *L*_decor_ aims to improve the compactness of descriptor by minimizing the correlation coefficients between nonidentical point descriptors within the same Siamese branch. The detailed calculation for *L*_rep_ and *L*_decor_ can be referred to [25].

Since the spatial relationship of feature point pairs is described by the complex mapping *𝒲*, the descriptor correspondence cannot be measured by linear operations. Inspired by the recent progress in contrastive learning of visual representation [30], we reinterpret the loss *L*_des_ as follows:

(10) $$\begin{array}{c} L_{des}=\underset{i}{\sum }-\log\frac{\exp\;\left(\text{sim}\left(f_{i}^{A},f_{j}^{B}\right)\right)}{\sum_{k}1_{\left[k!=j\right]\exp\;\left(\text{sim}\left(f_{i}^{A},f_{k}^{B}\right)\right)}}\text{,} \end{array}$$

with

(11) $$\begin{array}{c} \text{sim}\left(f_{i},f_{j}\right)=\frac{f_{i}^{⊤}\cdot f_{j}}{\tau \left\|f_{i}\right\|\cdot \left\|f_{j}\right\|}\text{,} \end{array}$$

where **f**_*i*_^*A*^ and **f**_*j*_^*B*^ denote the *i*-th and *j*-th descriptor predicted by branch *A* and *B*, respectively. Here, (**f**_*i*_^*A*^, **f**_*j*_^*B*^) is considered as a positive pair. The one-indicator 1_[*k*!=*j*]_ is only valid when *k* is not equal to *j*. Since there are 8 × 8 keypoints predicted for each image, a keypoint *i* on source image can only match one keypoint *j* on target image, while the rest 63 keypoints are considered as negatives for *i*. Thus, it ensures a nonzero denominator. The temperature *τ* is a hyperparameter, with a small value to reduce the impact of hard negative samples during the descriptor learning.

## 4. Experiment and Analysis

### 4.1. Experimental Setup

#### 4.1.1. Dataset

The proposed self-learning architecture for keypoint detection and matching is evaluated on the released FV set of the WoodScape fisheye data [29], which consists of 2037 training images and 442 test images collected by the fisheye camera installed on one vehicle. The camera's intrinsic and extrinsic parameters are also calibrated. Therefore, fisheye images can be undistorted through the image unwarping process introduced in Sec.  3.2. On the WoodScape dataset, the polynomial *ρ*(*θ*) in equation (10) is set with an order of *n*=4 with given coefficients *a*_1_ ~ *a*_4_.

#### 4.1.2. Implementation

The proposed self-learning architecture is implemented with PyTorch on a desktop with an Intel Xeon CPU of 2.5 GHz and an Nvidia 2080Ti GPU. The network is pretrained on the ordinary images in MS COCO dataset [31] and further trained on the WoodScape fisheye images. During the pretraining, ordinary homography transforms are utilized to generate paired images. In further training, a random mapping *𝒲* is applied for target fisheye image generation. The involved homography transform in mapping *𝒲* consists of scaling, rotation, and perspective transform, which are uniformly sampled with a margin of 0.1, *π*/2, and 0.1, respectively. The weights for loss terms are empirically set to *α*_rep_=1, *α*_pos_=1, *α*_score_=2, *α*_uni_=100, *α*_des_=0.001, and *α*_de cor_=0.03. We adopt the ADAM as the optimizer. The whole model is trained for ten epochs with data shuffling, a batch size of 16, and a learning rate of 0.000025. All images are resized to a uniform size of 240 × 320 pixels for processing efficiency.

#### 4.1.3. Metrics

The evaluation metrics adopted in experiments include the repeatability score (RS), the localization error (LE), the matching score (MS), and the homography accuracy (HA). The RS metric denotes the ratio between the number of points with correspondence and the total number of predicted points. A correspondence is established if points predicted from both images are located within the threshold *ε* = 3 by warping them into the same image plane. The LE metric is the mean distance between all matched point pairs according to the descriptors. The MS denotes the ratio between the number of good matches and the total number of points predicted in one image. A good match is defined as two corresponding points, which are also the nearest neighbors in descriptor space. To calculate HA, a source fisheye image is firstly unwarped by *ℱ*^−1^. The average distance between the image corners transformed by the estimated homography, and those transformed by the ground truth homography is calculated and defined as Homography error (HE). The HA is the ratio between the number of estimated homographies under a specified HE threshold (*ε* = 3) and the total number of homographies.

### 4.2. Exploration on Hyperparameter *τ*

The temperature parameter *τ* has a large impact on the descriptor correspondence loss *L*_des_. For hard negative samples, which can be easily classified as false positives, a smaller *τ* will reduce their weight during the learning. However, with an inappropriate small *τ*, true positives initialized with faraway positions can be neglected at the beginning of the training. To search for an appropriate temperature parameter, we train the network with different values of *τ*, and compare their test performance. The experimental results are reported in Table 2. As can be seen, with the setting of *τ*=0.05, the network achieves the best performance in terms of all metrics. Thus, we choose *τ*=0.05 as the optimal temperature parameter used in subsequent experiments.

### 4.3. Ablation Study on Model Setup

To verify the benefit of viewpoint transform (VT), deformable convolution (DC), and contrastive learning loss (CL), we conduct ablation studies on four different setups of the proposed network. The baseline (*B*) adopted in the experiment is the naive approach from work [25].

Test results are reported in Table 3. Obviously, by directly applying the baseline on fisheye images without viewpoint transform, the mean location error of corresponding points is relatively high, which is about 5 pixels and exceeds the default correspondence threshold (*ε*=3). Integrated with the viewpoint transform of fisheye images, the mean location error is reduced by about 2 pixels. The contrastive learning loss further yields a promotion on other metrics within the range of 0.18 to 0.24. With all setups, the proposed architecture achieves the best performance in terms of all metrics, demonstrating their improvements over the baseline.

### 4.4. Comparison with Nonlearning-Based Approaches

Here, we compare our architecture with other nonlearning-based keypoint approaches including SIFT, SURF, ORB, BRISK, KAZE, and AKAZE. Evaluation metrics are the same as in previous experiments. For SIFT, SURF, ORB, BRISK KAZE, and AKAZE, we directly use their implementation provided by OpenCV. To explore the performance of compared approaches under different challenging scenarios, we also add the following preprocessing operations to test images, respectively.

- Contrast change: random change in image brightness, saturation, and hue with up to 40%, 40%, and 20%, respectively
- Motion blur: blur filtering with a random filter size of up to 15 pixels
- Random noise: Gaussian noise with a variance randomly sampled from 30 to 70

For fairness, the viewpoint transform applied on one test image is the same across all scenarios. Test results are reported in Tables 4–6, respectively.

**Figure 7:**
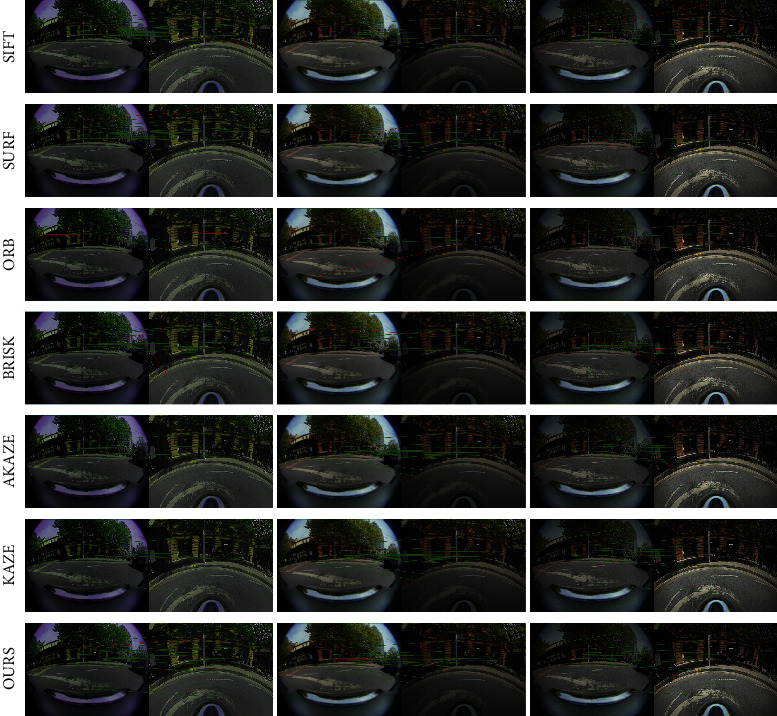
Examples of qualitative results in scenarios of contrast change (1st column), motion blur (2nd column), and noise (3rd column). Correct matches are linked by green lines while false matches are in red.

From the experimental results, it is obvious that our proposed approach achieves the best matching score and homography accuracy in scenarios with contrast change and motion blur. It also achieves comparable results with the top-ranked ORB and BRISK in terms of location error and repeatability score metrics. Additionally, it can be seen that the repeatability of the proposed approach is relative sensitive to noise. We assume that the image noise affects the keypoint selection in the proposed approach to some extent. However, it still achieves the second best on the metric of homography accuracy and matching score, only with minor gaps to the top-ranked SIFT. It is also noted that the proposed approach achieves a much smaller location error (second best) than SIFT. Test examples in different scenarios are shown in Figure 7. Considering the comprehensive performance, the proposed approach shows a relatively high robustness against contrast change, motion blur, and noise.

Furthermore, we present the feature detection and description time of evaluated keypoint models in Table 7. As can be seen, the ORB approach is the fastest among all handcrafted keypoint models, only requiring 0.06 second to process one frame. By running on the GPU platform, our proposed approach is also able to run in real time, with only 0.022 second per frame. Also, we calculate the value of FLOPs (floating point operations) and the number of parameters of our network, which are 7.4 G and 3.7 M, respectively, implying that our network is a relatively lightweight model.

## 5. Conclusions and Future Work

In this work, we propose a self-supervised learning architecture to address the challenging task of keypoint detection and matching on fisheye images. By integrating the viewpoint transform pipeline, the deformable convolution, and the contrastive learning loss, our method outperforms the baseline by a large margin. Through extensive experiments on challenging scenarios such as contrast change, motion blur, and noise, the comprehensive performance of the proposed approach is also demonstrated robust in terms of location error, homography accuracy, and matching score, compared to handcrafted models. As a direction of our future researches, we tend to integrate a more accurate and learnable undistortion scheme, which is free from the dependence on camera calibration parameters. Another direction is to include the multiscale image features to further improve the performance of the proposed approach.

## Figures and Tables

**Table 1:** Parameters of the keypoint network. “DConv” denotes the deformable convolution. All convolutional layers are followed by batch normalization and an activation function of leaky ReLU, except the last layer in each head.

	Module (kernel size)	Channel (in, out)	Stride
Backbone	2 × DConv (3 × 3)	(3, 32)	1
	1 × MaxPool (3 × 3)	(32, 32)	2
	2 × DConv (3 × 3)	(32, 64)	1
	1 × MaxPool (3 × 3)	(64, 64)	2
	2 × DConv (3 × 3)	(64, 128)	1
	1 × MaxPool (3 × 3)	(128, 128)	2
	2 × DConv (3 × 3)	(128, 256)	1
	1 × DConv (3 × 3)	(256, 128)	1
Head 1	1 × DConv (3 × 3)	(128, 256)	1
	1 × DConv (3 × 3)	(256, 1)	1
	1 × sigmoid	(1, 1)	1
Head 2	1 × DConv (3 × 3)	(128, 256)	1
	1 × DConv (3 × 3)	(256, 2)	1
	1 × sigmoid	(2, 2)	1
Head 3	1 × DConv (3 × 3)	(128, 256)	1
	1 × DConv (3 × 3)	(256, 256)	1

**Table 2:** Test results of networks trained by a different temperature parameter *τ*. An up-arrow indicates that higher values are better. The best values are denoted in bold.

Temperature	RS ↑	LE ↓	HA ↑	MS ↑
*τ*=0.03	0.33	2.76	0.39	0.36
*τ*=0.05	**0.35**	**2.73**	**0.42**	**0.39**
*τ*=0.1	0.31	2.75	0.37	0.35
*τ*=0.5	0.26	2.79	0.31	0.24

**Table 3:** Ablation study on different configurations of the proposed approach. The superscript ^*∗*^ denotes that the results are obtained at a threshold of 5 pixels. In the naive baseline, a hinge loss is adopted instead of the contrastive learning loss to learn descriptor correspondence. The best values are denoted in bold.

B	VT	CL	DC	RS ↑	LE ↓	HA ↑	MS ↑
✓				—	4.98^*∗*^	—	—
✓	✓			0.17	2.83	0.24	0.15
✓	✓	✓		0.35	2.73	0.37	0.39
✓	✓	✓	✓	0.41	2.53	0.41	0.43

**Table 4:** Test of keypoint models under contrast change. Best and second best are denoted in bold and italics.

Model	RS ↑	LE ↓	HA ↑	MS ↑
SIFT	0.40	3.57	0.24	0.32
SURF	0.39	3.78	*0.26*	*0.34*
ORB	*0.45*	**2.46**	0.17	0.16
BRISK	**0.48**	2.98	0.20	0.27
KAZE	0.37	2.79	0.27	0.35
AKAZE	0.35	2.93	0.19	0.20
Ours	0.43	*2.59*	**0.38**	**0.39**

**Table 5:** Test of keypoint models under motion blur. Best and second best are denoted in bold and italics.

Model	RS ↑	LE ↓	HA ↑	MS ↑
SIFT	0.42	3.61	0.23	*0.40*
SURF	0.43	3.85	*0.25*	0.39
ORB	0.40	**2.56**	0.09	0.09
BRISK	*0.44*	2.69	0.12	0.17
KAZE	0.36	2.98	0.22	0.29
AKAZE	0.33	3.03	0.10	0.14
Ours	**0.45**	*2.68*	**0.34**	**0.41**

**Table 6:** Test of keypoint models under noise. Best and second best are denoted in bold and italics.

Model	RS ↑	LE ↓	HA ↑	MS ↑
SIFT	*0.41*	3.49	**0.37**	**0.39**
SURF	0.39	3.77	0.31	0.31
ORB	0.40	**2.55**	0.15	0.11
BRISK	**0.43**	2.88	0.19	0.15
KAZE	0.34	2.65	0.23	0.24
AKAZE	0.28	2.78	0.11	0.17
Ours	0.33	*2.61*	*0.33*	*0.32*

**Table 7:** Feature detection and description time of compared keypoint models. Superscript ^*∗*^ denotes utilization of GPU.

Model	SIFT	SURF	ORB	BRISK	KAZE	AKAZE	Ours^*∗*^
Time (s)	0.2	0.18	0.06	0.27	0.4	0.072	**0.022**

## Data Availability

All the data are available in the article.
